# Online Decision Support for Implementing Evidence-Based HPV Vaccination Strategies in Texas Safety-Net Pediatric Clinics: Impact on HPV, MCV, and Tdap Initiation

**DOI:** 10.3390/healthcare14040519

**Published:** 2026-02-18

**Authors:** Ross Shegog, Hanxiao Sun, Erica L. Frost, Laura C. Thormaehlen, Travis A. Teague, Catherine Mary Healy, Hina Azam, Aadeel Khawaja, Laura Aubree Shay, Dale S. Mantey, Sally W. Vernon, Lara S. Savas

**Affiliations:** 1 Department of Health Promotion and Behavioral Sciences, University of Texas School of Public Health, Houston, TX 77030, USAerica.l.frost@uth.tmc.edu (E.L.F.); travis.a.teague@uth.tmc.edu (T.A.T.);; 2 Department of Pediatrics, Infectious Diseases Section, Baylor College of Medicine, Houston, TX 77030, USA; 3 Ibn Sina Foundation, Houston, TX 77053, USA

**Keywords:** HPV, MCV4, Tdap, vaccination, pediatric, decision support, implementation, digital health, usability, feasibility

## Abstract

**Introduction**: HPV vaccination rates for adolescents in the United States are below recommended levels. The Adolescent Vaccination Program (AVP) guides pediatric clinics on how to implement evidence-based strategies to increase HPV vaccination rates. These strategies comprise the adoption of (1) immunization champions, (2) provider assessment and feedback, (3) continuing education, and (4) prompts, (5) parent reminders, and (6) parent education. The AVP systems-based intervention has demonstrated increased HPV vaccination rates in large urban pediatric clinic networks. The purpose of this study was to assess the feasibility of using an online decision support tool, the AVP Implementation Tool (AVP-IT), to implement AVP strategies in safety-net clinics to improve healthcare for the medically underserved in Texas. **Methods**: AVP immunization clinic staff champions in four urban safety-net clinics completed tailored Action Plans within the AVP-IT to guide strategy implementation, received webinar training from the research team commensurate to each AVP strategy, and participated in monthly monitoring calls with AVP-IT project staff over a 33-month period from 2022 to 2024. **Results**: All clinics made progress toward full implementation of AVP strategies. Interrupted time series (ITS) trend analysis demonstrated that AVP-IT implementation was associated with an immediate boost in HPV vaccine initiation rates (*p* < 0.001) and that long-term trends (ITS slopes) were significant for HPV, MCV4, and Tdap vaccines despite low post-COVID-19 pandemic rates (*p* < 0.001). Vaccination rates using raw data (mean differences) were not longitudinally significant except for older youth aged 13–17 years. **Conclusions**: The AVP-IT promises accessible and practical decision support to implement strategies to increase HPV vaccination rates in safety-net clinics. Scale-up in these clinics will require leadership support, technical assistance, and EHR optimization.

## 1. Introduction

Human papillomavirus (HPV) infection prevalence is approaching 79 million people in the United States (U.S.), with an incidence of 14 million newly infected individuals annually [1]. HPV infection (predominantly high-risk types 16 and 18) is associated with a majority of cervical and anal cancers, oropharyngeal cancers, vaginal and vulvar cancers, and penile cancers (all > 60%) [2,3,4,5,6,7]. Treatment of these cancers contributes to an increased healthcare cost burden [8]. The HPV vaccine is associated with preventing HPV infection, precancerous lesions, and genital warts [9,10,11,12]. The initiation of the HPV vaccination series is therefore recommended for all children aged 11 to 12 years [13,14] and, more recently, for children aged 9–10 years [15,16,17,18,19,20,21,22]. The HPV vaccine is safe [23,24] and effective [10]. Despite recommendations, HPV vaccination rates have yet to reach the U.S. Healthy People 2030 goal of 80% for series completion in adolescents aged 13 to 15 years [25]. Rates are also below those of other routinely administered adolescent vaccines recommended for this age group: the meningococcal conjugate quadrivalent (MCV4) and diphtheria, tetanus, and acellular pertussis (Tdap) vaccines [26].

Strategies to increase HPV vaccination rates within a systems-based implementation (inclusive of organizational, provider, and patient levels) have evidence of effectiveness [27,28,29,30,31]. Such strategies include providing assessment and feedback to healthcare providers (HCPs) [27] to ensure HCPs are prompted to vaccinate eligible youth in real time during clinic visits [28], to issue parents with reminders regarding their child’s vaccine eligibility [29], and to provide HCPs and parents with education in concert with these other healthcare system-based strategies [30]. Despite the evidence of their effectiveness, the adoption and implementation of these strategies between clinics and within clinic networks have been inconsistent [31,32,33].

The Adolescent Vaccination Program (AVP) is a multi-component, systems-based intervention that promotes evidence-based strategies to increase HPV vaccination rates in pediatric clinics for youth aged 11–17 years [32]. These strategies comprise (1) the adoption of immunization champions within each clinic, (2) providing HCPs with assessment and feedback reports on their HPV vaccination rates and missed opportunities, (3) continuing medical or nursing education credits (CME/CNE) focused on HPV and HPV vaccination, (4) real-time prompts alerting HCPs of the presence of vaccine-eligible youth during the clinic visit, (5) reminders to vaccinate provided to parents of vaccine-eligible youth, and (6) HPV education for parents to mitigate their vaccine hesitancy. AVP implementation has resulted in significantly increased HPV initiation and series completion in large urban pediatric networks in Houston and San Antonio, Texas [33,34,35,36].

Demonstrating the effectiveness of strategies to increase HPV vaccination rates is insufficient to ensure their broad-based adoption and implementation. However, strategies exist to support the implementation of evidence-based clinical practices [37]. Dissemination researchers have recommended the utility of online decision support tools to translate evidence-based science into practice [38]. This approach has aided decision making in healthcare settings by enhancing drug dosing, preventive care, and active medical care [39,40,41]. Theoretically and empirically based online decision support could assist in guiding HCPs and clinic managers to adopt and implement evidence-based strategies to increase HPV vaccination.

**Figure 1:**
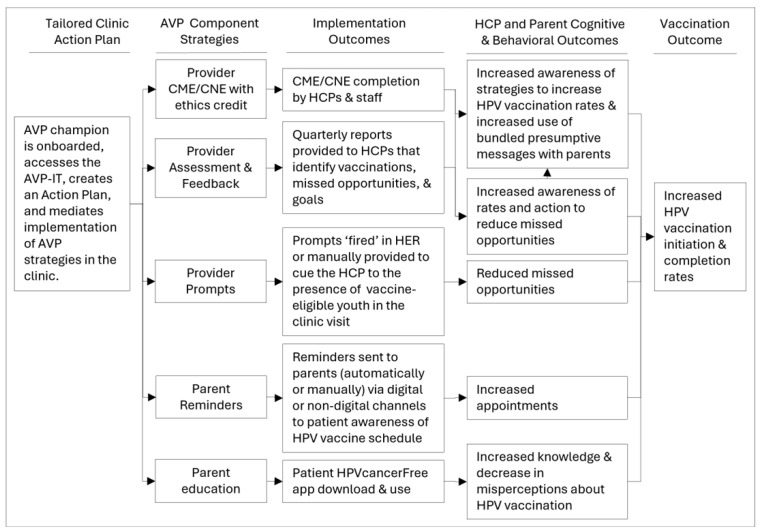
Logic Model of the AVP Action Plan.

The efficacy of the AVP and decision support suggests that adapting the AVP for online decision support could enable clinics to independently implement evidence-based strategies in a form that is scalable for broad geographic reach. Pursuant to this, an online decision support tool, the AVP Implementation Tool (AVP-IT), was developed to guide clinics in implementing the AVP through the use of a tailored Action Plan dynamically generated within the tool (Figure 1).

Individual and organizational factors have been identified that facilitate the implementation of decision support tools [42,43]. Individuals must perceive the tool as acceptable, credible, easy, simple, beneficial, impactful, understandable, and appealing [44] and organizations must have the capacity to support implementation, inclusive of leadership and managerial support, and resource commitment [45]. Usability and feasibility testing of the AVP-IT prototype was conducted as an important formative step to ensure functionality and acceptability in a clinic setting [46]. Five HCPs rated the AVP-IT favorably on acceptability, credibility, ease, helpfulness, impact, and appeal, exceeding the a priori criteria for a minimum of 80% agreement, and rated it as providing an easier and more effective approach to implement HPV vaccination strategies than their usual practice (*p* < 0.05) [46]. The AVP-IT facilitated increased strategy implementation by 3-month follow-up [46]. These findings indicated the utility of further feasibility testing.

Federally qualified health clinics (FQHCs) and safety-net clinics provide community-based comprehensive primary, dental, and mental health services to care for medically underserved populations (e.g., uninsured and low-income communities) with a high burden of chronic illness, regardless of their ability to pay [47,48]. They provide an important medical home for underserved adolescents and have tremendous potential to address disparities in preventable diseases [49].

In the U.S., costs can vary from $250 to $350 (USD) for the full HPV vaccine series [50]. This can represent a financial burden, but this cost is often covered by insurance for adults of 26 years of age and under [50], and publicly funded programs provide free or low-cost vaccines to low-income children. In Texas, the HPV vaccine (Gardasil 9) is typically free for youth under the age of 18 years from the Vaccine for Children (VFC) program but, after 18 years, cost becomes and important barrier to HPV vaccination among the majority of uninsured adults [50]. Despite cost offsets, challenges to HPV vaccine delivery can include residual cost barriers (e.g., administration fees) and factors related to dosing schedules, target population, and parental ambivalence [50,51].

In the U.S., the states of Rhode Island and Virginia, the District of Columbia, and Puerto Rico require vaccination for school admission, but HPV vaccination is not universally mandated [52]. Parents can usually opt out of mandated vaccination programs, and school requirements in the states of Louisiana, Arizona, West Virginia, and South Carolina specifically exclude HPV vaccination. The state of Texas mandates MCV and Tdap vaccinations for youth aged 11–12 years for school admission, but HPV vaccination is not mandated. Given this, there is a need for effective implementation of comprehensive evidence-based strategies to increase HPV vaccination to ensure that disadvantaged youth are vaccinated in non-profit safety-net clinics [49,53,54,55,56].

The purpose of this study was to assess the feasibility and impact of using the AVP-IT digital decision support to guide implementation of evidence-based strategies to increase HPV vaccination rates, as well as mandated MCV4 and Tdap adolescent vaccines, to improve healthcare in community safety-net clinic settings. Primary outcomes were rates of HPV vaccine initiation, secondary outcomes were MCV4 and Tdap vaccine initiation rates, and tertiary outcomes were implementation fidelity metrics of strategies and tasks completed by AVP champions. This study was conducted from March 2022 to November 2024. A historical AVP-IT pre-implementation counterpart, from April 2020 to March 2022, was included for longitudinal trend analysis to capture potential transitions in vaccination rates. This duration was coincidently embedded within the COVID-19 pandemic (March 2020 to May 2023), which offered additional analytic opportunity to examine the impact of COVID-19 on longitudinal vaccination rates [57]. This study follows a precedent in the use of interrupted time series (ITS) designs within FQHCs and safety-net clinics for longitudinal evaluation of programs. Segmented regression has been applied on longitudinal electronic health record (EHR) data to analyze changes in trends before and after intervention implementation to evaluate quality improvement, cancer screening, and care equity [58,59,60]. ITS is a rigorous quasi-experimental approach that can control for secular trends and evaluate the impact of policies or programs over time [60]. This study contributes to understanding the application of using online decision support to improve primary healthcare for uninsured and low-income families.

## 2. Materials and Methods

### 2.1. Study Design and Setting

A single-group pre–posttest design was conducted in five clinics within a safety-net clinic network in the Greater Houston area, each comprising one to three HCPs. The clinics provided diagnostic, medical, specialty, immunization, and behavioral health services to clinically underserved populations. The patient population was Hispanic (43–67%), African American (14–32%), and South Asian (11–19%) and mostly uninsured or paying out-of-pocket for healthcare services (74–94%). This study was conducted in Houston, Texas, USA, between March 2022 and November 2024 (IRB #HSC-SPH-22-0204).

### 2.2. The AVP-IT

**Figure 2:**
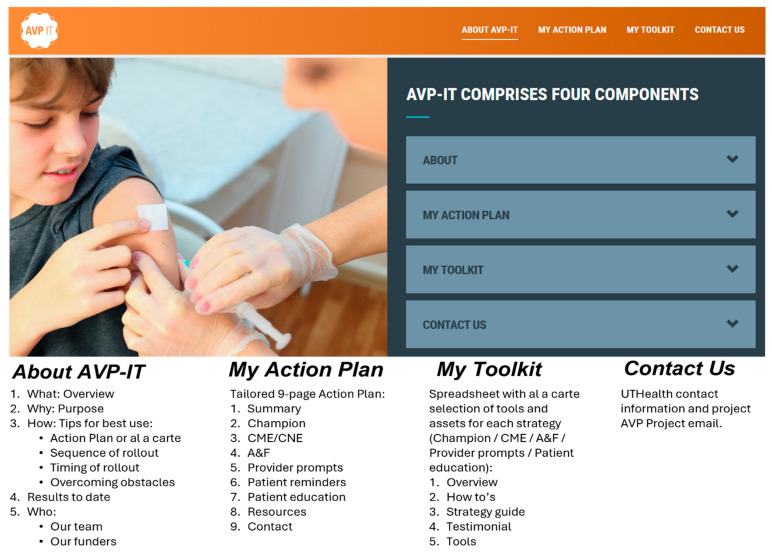
AVP components.

The Adolescent Vaccination Program Implementation Tool (AVP-IT) is a decision support website designed to assist pediatric clinic staff to adopt, implement, and maintain evidence-based HPV vaccination strategies within their clinic (Figure 2). It guides staff to (1) assess their clinic’s readiness to implement each AVP strategy, (2) tailor implementation strategies to fit clinics of any size and type, and (3) increase systems knowledge and capacity to improve low HPV vaccination rates. The development and formative evaluation of the AVP-IT have been described elsewhere [46]. The AVP-IT website is housed on a secure UTHealth server and is accessible online to any clinic (https://avp.sph.uth.edu).

**Figure 3:**
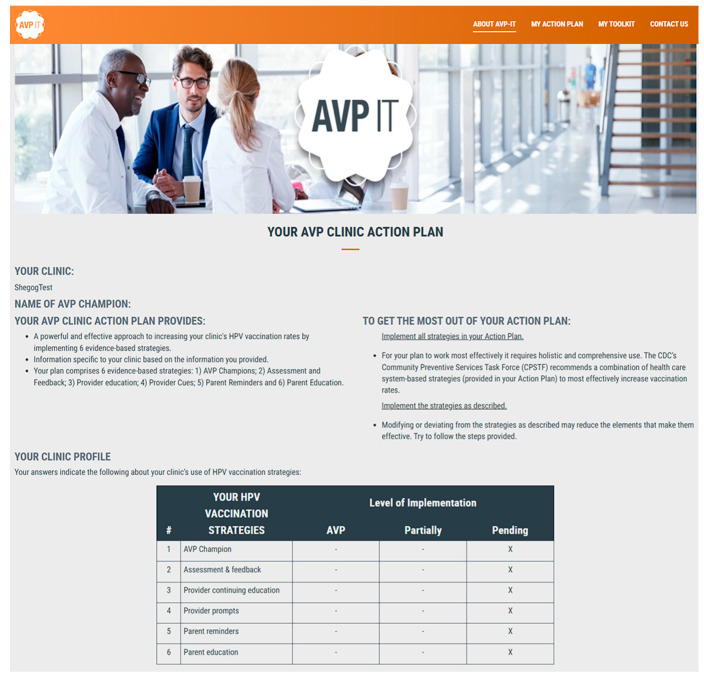
Clinic AVP-IT Action Plan (page 1) with assessment table demarcating (with an ‘x’) the current implementation status of each strategy.

AVP champions used the AVP-IT by visiting each of four website pages on the AVP-IT website. Page 1, ‘About AVP-IT,’ provided an overview and background on the AVP-IT in video and text with links to research publications. Page 2 of the AVP-IT, ‘My Action Plan,’ included an Action Plan Wizard that guides the AVP champion to enter descriptive data about their clinic, including the current level of implementation for each of the AVP strategies, by responding to multiple-choice questions. Based on these responses, the AVP-IT dynamically generated a printable Action Plan tailored to the clinic’s specific characteristics and needs. The Action Plan is a guide on how to implement the HPV vaccination strategies of linking HCPs to continuing medical and nursing education (CME/CNE), generating a quarterly assessment and feedback report, initiating patient reminders, instituting electronic health record (EHR) real-time prompts regarding vaccine-eligible youth, and linking parents to the HPVcancerFree parent education app (Figure 3) [32,34]. The Action Plan provided (1) a definition of each strategy, (2) ‘How To steps’ for implementation, (3) ‘Tools’ (resources to facilitate implementation), (4) ‘Tips for Success’ (bulleted practical recommendations informed by previous studies), and (5) ‘Quotes from the Field’ (tips from successful implementors). Page 3 of the AVP-IT, ‘My Toolkit,’ provided the AVP champion with an ‘à la carte’ selection of resources comprising video testimonials on the importance of each strategy and tips on successful implementation, assessment and feedback report templates, algorithms and script for EHR-based provider and patient reminders, and CME/CNE and HPVcancerFree app promotional flyers. Page 4 of the AVP-IT, ‘Contact Us,’ provided the AVP champion with a form to contact the AVP research team with questions or to report issues with the AVP-IT website. Champions received four webinar trainings on the rollout of Adolescent Vaccination Program (AVP) strategies prior to implementation and participated in monthly monitoring calls with AVP-IT project staff.

### 2.3. Feasibility Testing

Clinic directors selected an AVP champion for each clinic to take responsibility for creating and implementing their clinic’s Action Plan. Champions could include chief medical officers, HCPs, clinic administrators, practice managers, or clinic staff. The champions were provided with an email with instructions for the feasibility trial, accessed the AVP-IT website, input data into the Action Plan Wizard, printed their clinic’s tailored Action Plan, and were instructed to complete the Action Plan steps to implement the AVP strategies in their clinic. Implementation status was assessed at baseline and at the completion of the study using data reported by AVP champions in the Wizard. These data confirmed the degree of fidelity of use by categorizing each strategy as ‘pending,’ ‘partially implemented,’ or ‘fully implemented.’ Strategies were ‘fully implemented’ if they were fully adherent to the AVP-IT Action Plan criteria, ‘partially implemented’ if they did not meet the full criteria, and ‘pending’ if they were not yet initiated in the clinic. Monthly monitoring calls were conducted with the AVP champion to update research staff on implementation status, identify facilitators, and troubleshoot barriers. A behavioral inventory checklist was developed that provided a series of enabling behaviors to complete each strategy. This became a reference point for monthly monitoring calls.

### 2.4. Measurement

#### 2.4.1. Provider and Patient Demographics

AVP champions, clinic managers, and pediatricians completed a demographic survey (i.e., job title, age, gender, education level, and race/ethnicity) at the commencement of the study. Patient sample demographics were drawn from available cumulative EHR data comprising age, clinic visits, gender, race, and insurance status.

#### 2.4.2. AVP Champion Implementation Task Fidelity

The fidelity of task performance to implement AVP strategies was assessed using task checklists completed during monthly monitoring calls between the champions and research staff. Tasks were designed to enable champions to raise provider awareness of the CME/CNE with ethics credit and link them to it (n = 9), ensure providers were prompted regarding a youth’s vaccine eligibility during clinic visits (n = 2), ensure parents of vaccine-eligible youth were reminded to initiate or complete vaccinations (n = 9), and raise the awareness of parents regarding the HPVcancerFree app and link them to it (n = 5). These tasks are listed in the Results Section.

#### 2.4.3. AVP-IT Strategy Implementation

AVP-IT strategy implementation was assessed using data provided by the clinic AVP champion when creating their tailored clinic Action Plan at the commencement and closure of the study. The degree of implementation of each strategy was rated as ‘pending’, ‘partially implemented’, and ‘fully implemented’. The prompts used to create the tailored Action Plan are listed in the Results Section.

#### 2.4.4. Vaccination Initiation

Initiation rates for HPV vaccinations with 4-valent and 9-valent HPV vaccines were used to evaluate the impact of the AVP-IT. Initiation rates represented the proportion of individuals who received any HPV vaccine dose. To account for the multi-dose nature of the vaccine, rates among previously unvaccinated populations were assessed. Two single-dose state-mandatory vaccinations, the meningococcal conjugate vaccine (MCV4) and the tetanus, diphtheria, and acellular pertussis (Tdap) vaccine, were also assessed to provide comparison in downstream analysis.

Vaccination data were collected and analyzed for youth aged 11–17 years from April 2020 to March 2024, including a two-year pre-implementation period for comparative analysis. Protocols were developed to extract vaccination records from the NextGen EHR database used in the participating clinics. These comprised the use of a built-in keyword search engine and use of a customized workflow to process Health Level Seven (HL7) messages based on vaccine administration (CVX) codes (HPV vaccine: 62, 165; MCV4: 114, 136; Tdap: 115) [61,62]. All identified cases were cross-validated by checking Date of Service, Administered Clinic Location, and Patient Chart Number. Duplicate records and entries with missing information were removed. For repeatedly visiting patients, vaccinated individuals were counted once, while unvaccinated individuals were counted only at their first visit within each semester.

Population-level rates were calculated quarterly (Q1: January–March, Q2: April–June, Q3: July–September, Q4: October–December) and standardized as proportions among vaccine-naive youth observed in each quarter. A pretest–posttest analysis was conducted to evaluate the effectiveness of AVP-IT. Youth were further stratified into two age subgroups targeted by the AVP-IT, 11-12 and 13-17 years old, to examine if AVP-IT effects differ across age groups.

### 2.5. Analysis

#### 2.5.1. Demographic and Implementation Assessments

Analysis of provider and sample demographics comprised descriptive statistics, and *t*-test and chi-square assessment, for sample pre- and post-implementation. Assessment of AVP champion fidelity and AVP strategy implementation was descriptive.

#### 2.5.2. Vaccination Efficacy Assessment

Descriptive statistics were first evaluated. Paired *t*-tests were performed to examine changes in raw vaccination volume and initiation rates before and after AVP-IT implementation, and among age subgroups. An interrupted time series (ITS) analysis was used due to the lack of a simultaneous comparison population without AVP-IT exposure. ITS is a widely used quasi-experimental approach for evaluating population-level health interventions by self-comparing outcome trends before and after implementation. ITS has been successfully applied in prior vaccine and policy evaluations, including assessments of pneumococcal conjugate vaccine impact, and changes in Medicaid programs [63,64]. Using this framework, estimations were made of both the immediate change in vaccination initiation rates following AVP-IT implementation and subsequent changes in temporal trends. This allowed for assessment of short-term intervention effects and sustained impacts over time. The generalized formula (below) was implemented on time-series-based initiation rates, using the Linear and Nonlinear Mixed Effects Models (nlme) R package (Version 3.1-166) [64,65]:

$$Y_{t}=\;\beta_{0}+\beta_{1}\times time_{t}+\beta_{2}\times intervention_{t}+\beta_{3}\times time\;after\;intervention_{t}+\beta_{c}\times covariates_{t}+\epsilon_{t}$$

where *covariates_t_* = {quarterly indicators, the number of youth with COVID-19-related visits, time after distributing COVID-19 vaccines, indicators of COVID-19 outbreaks in Texas}. Here $Y_{t}$ referred to the mean vaccination rate of interest in quarter t. The model included three time-related variables: (1) the longitudinal time trend that captures the consistent long-term natural vaccination trend counting consecutive quarters from Quarter 2 (April–June), 2020 (pre-implementation), through AVP-IT implementation until Quarter 1 (Jan–March), 2024; (2) intervention_t_ as a binary indicator of AVP-IT implementation status; and (3) the post-implementation time trend counting consecutive quarters from AVP-IT implementation since Quarter 2 (April–June), 2022, through to Quarter 1 (Jan–March), 2024. The coefficients represented mean vaccination rates on different scales: *β*_0_ for baseline vaccination level, *β*_1_ for average natural rate changes for an estimated long-lasting vaccination trend independent of AVP-IT implementation, *β*_2_ for average immediate changes after implementation, and *β*_3_ for average rate changes in post-implementation for a long-term trend estimate.

Prior to model fitting, we examined the autocorrelation structure of vaccination initiation rates to account for multi-dose administration schedules and commonly observed seasonal fluctuations [65]. Autocorrelation plots were used to assess lagged dependence using the R default stats package (Version 3.6.2). Logarithmic transformation was applied when necessary to ensure invertibility of the covariance matrix. Quarterly indicator variables and, when indicated, fourth-order autoregressive residual structures were incorporated to capture seasonal patterns and lagged effects within the regression framework. Three covariates were included as needed to maximumly adjust for the impact of the COVID-19 pandemic: (1) the number of patients with COVID-19-related visits, denoting the impact from overwhelming population flow, (2) a binary variable indicating the availability of COVID-19 vaccines, and (3) a binary variable accounting for high COVID-19 rates during the outbreak, denoting the high inpatient and medical resource burden, when Texas’s 7-day cumulative cases exceeded 20 thousand [66]. We performed forward feature selection to include as many significant features as possible, while keeping the standard error of residuals as small as possible. For model parsimony, all insignificant variables were excluded. We further performed model diagnostics by examining distributions of residuals, including by quarter as needed. Age-subgroup analyses were conducted comparing early (11 to 12 years old) and middle-to-late (13 to 17) patients to identify potential effect heterogeneity. The model details are shown in the Results Section and in the Supplementary Materials). Interested readers can request summary statistics and code via GitHub upon request to the authors [67].

## 3. Results

### 3.1. AVP-IT Champion and Youth Sample Demographics

**Figure 4:**
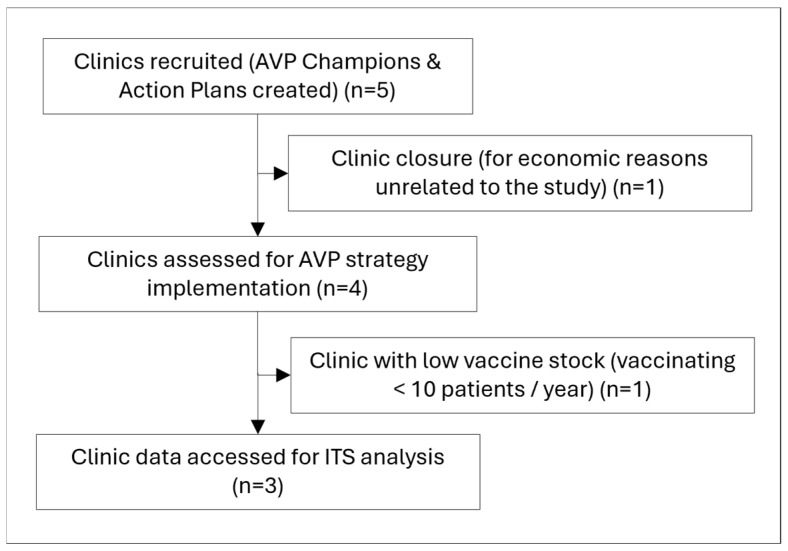
Clinic recruitment and inclusion/exclusion.

AVP-IT champions were successfully identified at each clinic to serve as the primary points of contact for their practice. They comprised medical assistants and were female, Hispanic, and between the ages of 20 and 29, and reported their highest level of education as a college degree (n = 3) or some college (n = 1). Youth (n = 9549) aged 11–17 years were observed between April 2020 and March 2024, with 5702 and 4928 identified before and after AVP-IT implementation, respectively (Table 1). Table 1 reports a subsample with available demographic data from 4351 pre-implementation and 3943 post-implementation patients, respectively. During the study, one clinic site closed for economic reasons unrelated to the study (Figure 4). Data from one of the remaining four clinics were excluded due to low vaccination stock, having less than 10 target vaccinations administered per year. Decreases in average patient age and COVID-19 testing coverage (alongside an increasing proportion of self-pay patients) were observed across study periods. No significant changes were observed for average clinic visits, gender, or race.

### 3.2. AVP-IT Champion Implementation Task Fidelity

Champions reported a task completion rate of 25-100% for each of the 25 tasks (Table 2). Provider continuing education: Between 44 and 89% of tasks were completed across the clinics to link providers to the CME/CNE. This included distributing and displaying the AVP CME/CNE promotional flyer and notifying clinic staff about the AVP CME/CNE in person and through email. All champions reported completing the AVP CME/CNE. Provider prompts: All champions reported reviewing HPV vaccine eligibility with their providers and medical assistants, and that their clinic routinely checked HPV vaccination eligibility (in the EHR and ImmTrac) for all youth attending well-child visits. Half reported making notes in patients’ charts to alert providers of vaccine-eligible youth. These tasks were conducted manually, without optimization of the EHR for automatic alert functions. Parent reminders: Between 44 and 78% of tasks were completed across the clinics. All champions reported reviewing parent reminder best practices with their HCPs and medical assistants and applying decision rules to determine youth vaccine eligibility. Most (75%) reported reviewing and updating youth contact information, scheduling a follow-up visit before the youth leaves the clinic, and logging this next visit date. Half the clinics reported that their medical assistants routinely provide parents with ImmTrac (state vaccine registry) recall letters that include dates the youth is due for their follow-up dose(s) or assigning a staff member to send reminders. Parent education: Between 25 and 80% of tasks were completed across the clinics. Most champions reported hanging the HPVcancerFree app flyer and posters in their clinic’s waiting and exam rooms (75–100%) and distributing the flyer to clinic staff and in meetings (75%). Half reported providing copies of the flyer to parents of eligible youth during appointments.

### 3.3. AVP-IT Strategy Implementation Outcomes

Each champion completed the AVP-IT Action Plan Wizard. At baseline, implementation of AVP strategies was skewed towards a classification of ‘pending’ across the five clinic sites (Table 3). Sites mainly rated themselves as ‘pending’ for assessment and feedback (n = 3), provider prompts (n = 4), and parent reminders (n = 3), and as ‘partially implemented’ for continuing education (n = 4) and parent reminders (n = 4). At 33-month follow-up, implementation of AVP strategies was skewed towards ratings of ‘fully implemented’ across the four clinic sites. Sites mainly rated themselves as ‘fully implemented’ for AVP champions (n = 3), CME/CNE completion (n = 2), parent reminders (n = 4), and parent education (n = 4). Assessment and feedback were split between ‘partial’ and ‘fully’ implemented (both n = 2), while provider prompts remained skewed to ‘pending’ (n = 3).

### 3.4. Vaccination Frequency Pre–Post-AVP-IT Implementation for HPV, MCV4, and Tdap

Prior to AVP-IT implementation, 593 eligible patients received a total of 652 HPV vaccine doses. Following AVP-IT implementation 475 eligible youth received a total of 519 doses. Similarly, for the MCV4 vaccine, prior to AVP-IT implementation 1000 eligible youth received 1011 doses, and following AVP-IT implementation 1447 patients received 1485 doses. This included second (n = 47) or third (n = 1) doses of MCV4 for youth with particular health conditions [66]. For the Tdap vaccine, prior to implementation 942 eligible patients received 993 doses, and following implementation 1412 patients received 1589 doses. This included second (n = 173), third (n = 26), and fourth (n = 1) doses of Tdap for youth with particular health needs (e.g., deep lacerations).

### 3.5. Quarterly Trends in Vaccine Amount and Initiation Rates

**Figure 5:**
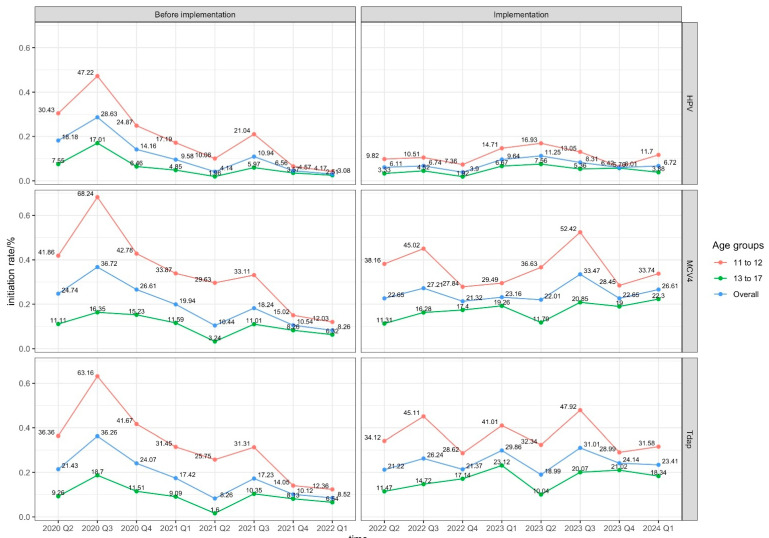
Quarterly trends in vaccine initiation rates.

A consistent seasonal pattern existed for all three vaccines throughout the observation period, with greater volume in the third quarter (summer) and lower volume in the first and second quarters (winter). This aligned with the school calendar based on annual vaccination requirements for school registration, as was detected in the autocorrelation plots (Supplementary Figures S1–S4). A significant patient-visiting surge occurred during the COVID-19 outbreak in 2021–2022. Standardized vaccine initiation rates therefore showed substantial variation, with a significant suppression during the COVID-19 outbreak because of this surge. However, initiation rates for MCV4 and Tdap resumed to pre-pandemic levels in 2024, while HPV vaccine initiation rates remained low. No significant changes in quarterly initiation rates were observed for the three vaccines after AVP-IT implementation. The concrete age-stratified initiation rates are also plotted in Figure 5.

**Figure 6:**
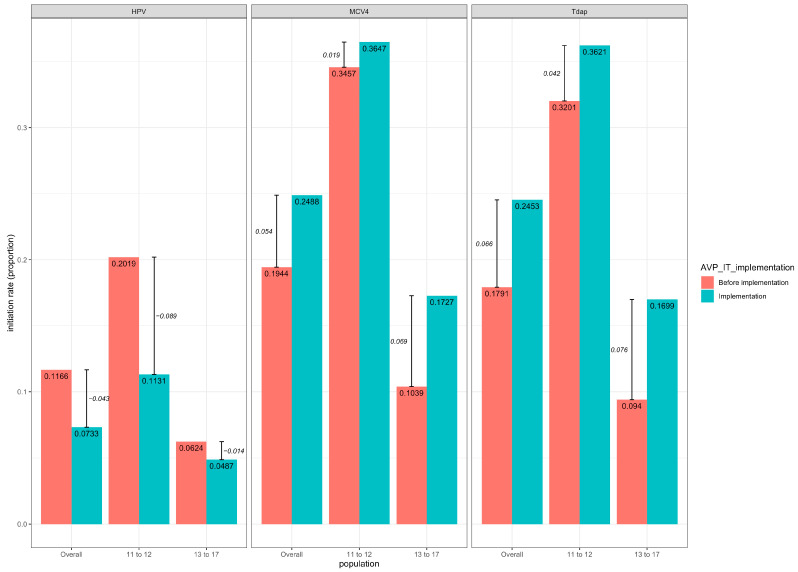
Changes in mean initiation rates of HPV, MCV, and Tdap vaccines pre- and post-AVP-IT implementation. The y-axis stands for the average initiation rates for each age group. The mean initiation rate differences before and after AVP-IT implementation are annotated in italics.

Raw HPV vaccination volumes had a slight insignificant decline with a mean decrease of 14.63 doses (95% CI: −16.62–45.87, *p* = 0.305). In contrast, statistically significant increases were observed in the number of patients receiving MCV4 and Tdap vaccines after AVP-IT implementation, with mean quarterly increases of 57.75 doses (95% CI: 15.37–100.13, *p* < 0.05) and 60.00 doses (95% CI: 17.80–102.20, *p* < 0.05), respectively. However, no significant changes in initiation rates were observed for any of the three vaccines after AVP-IT implementation. Mean quarterly changes in vaccinated proportions among vaccine-eligible patients were −0.04 (95% CI: −0.04–0.12, *p* = 0.25) for HPV, 0.05 (95% CI: −0.03–0.14, *p* = 0.18) for MCV4, and 0.07 (95% CI: −0.01–0.15, *p* = 0.09) for Tdap, despite significant increases in older (13–17 years) youth for MCV4 (0.07, 95% CI: 0.02–0.12) and Tdap (0.08, 95% CI: 0.03–0.13) (Figure 6)**.**

#### Age Variations for Vaccine Initiation Rates

**Figure 7:**
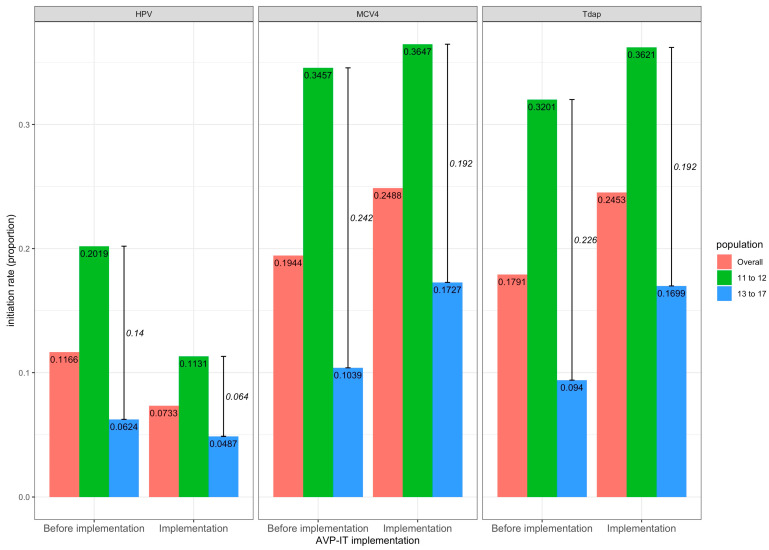
Mean initiation rate differences for HPV, MCV, and Tdap vaccines by age. The y-axis stands for the average initiation rates before and after AVP-IT implementation. The mean across-age-group initiation rate differences are annotated in italics.

Pre-implementation subgroup analysis revealed higher vaccination rates among younger (11–12 years) compared to older (13–17 years) youth across all three vaccines (Figure 7), with mean differences of 0.14 (95% CI: 0.06–0.22, *p* < 0.01) for HPV, 0.24 (95% CI: 0.12–0.36, *p* < 0.01) for MCV4, and 0.22 (95% CI: 0.12–0.33, *p* < 0.01) for Tdap. This gap narrowed after AVP-IT implementation, with post-implementation differences of 0.06 (95% CI: 0.04–0.09, *p* < 0.01) for HPV, 0.19 (95% CI: 0.11–0.27, *p* < 0.01) for MCV4, and 0.19 (95% CI: 0.13-0.26, *p* < 0.001) for Tdap.

### 3.6. Effectiveness of AVP-IT Implementation

#### 3.6.1. HPV Initiation Rate Modeling

**Figure 8:**
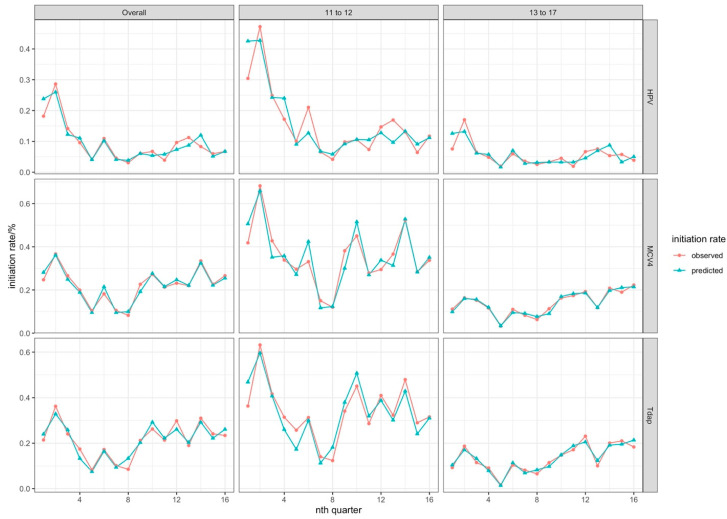
The relationship between observed and predicted vaccination rates.

**Figure 9:**
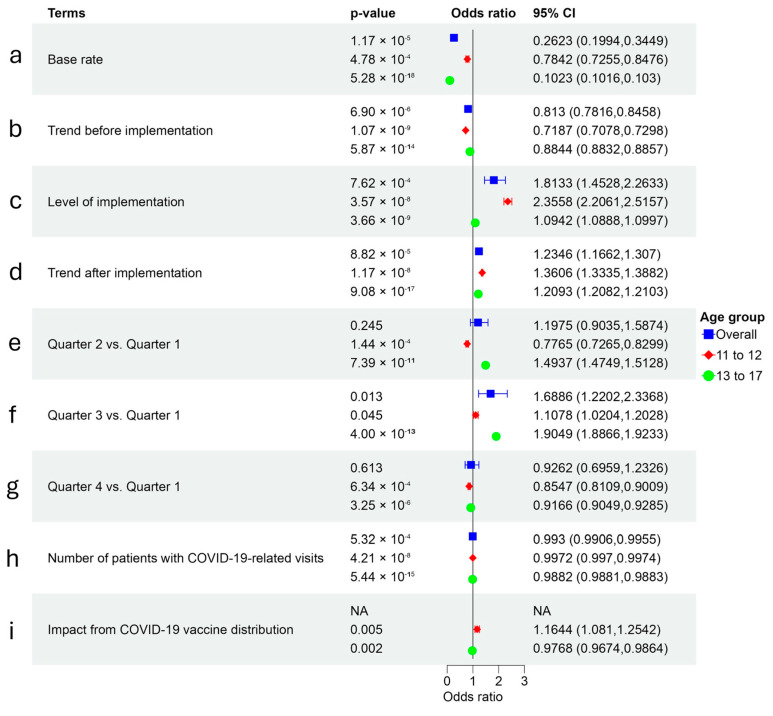
AVP-IT effectiveness—HPV initiation rate modeling.

In the ITS modeling, logarithmic-transformed HPV initiation rates were significantly associated with both pre- and post-implementation vaccination trends, AVP-IT implementation, seasonality, and the COVID-19 pandemic, which were impactful from Quarter 2, 2020 to Quarter 1, 2024. The summary of modeling elements is provided in Table 4, the association between observed and predicted vaccination rates in Figure 8, and the AVP-IT effectiveness in Figure 9. The baseline rate indicated that 26.23% (95% CI: 19.94–34.49%, *p* < 0.001) of eligible youth received at least one HPV vaccine dose in the first quarter with a decreasing trend over time to 18.7% (95% CI: 15.42–21.84) or 0.81 times per quarter (95% CI: 0.72–0.88, *p* < 0.001), and assuming no effects from any other factors (Figure 9a,b). However, AVP-IT implementation significantly increased the likelihood of HPV vaccination administration by as much as 1.81 times (95% CI: 1.45–2.26, *p* < 0.001) (Figure 9c) and fostered a long-term positive trend, after adjusting for natural trend, quarter, and COVID-19 impacts (1.23 times per quarter, 95% CI: 1.17–1.31, *p* < 0.001) (Figure 9d).

#### 3.6.2. Regular Seasonal Patterns and Temporal COVID-19 Effects

Regular seasonal patterns and temporal COVID-19 effects were significant, with HPV initiation rates varying by quarter. Youth were 1.69 times more likely to receive vaccination in the third quarter (95% CI: 1.22–2.34, *p* < 0.05) compared to the first quarter (Figure 9f). Temporal COVID-19 effects were also observed. The COVID-19 pandemic significantly reduced the likelihood of HPV vaccination, with each additional COVID-19-related visit lowering the likelihood of HPV vaccination by 0.007 times (95% CI: 0.005–0.009, *p* < 0.001).

#### 3.6.3. Age-Specific Effects

Age-specific effects were also significant. Younger youth demonstrated a stronger decreasing baseline trend (0.72 times, 95% CI: 0.71–0.73, *p* < 0.001) compared to older youth (0.88 times, 95% CI: 0.88–0.89, *p* < 0.001) (Figure 9b). However, younger youth were more responsive to AVP-IT strategies, with stronger implementation (2.36 times, 95% CI: 2.21–2.52, *p* < 0.001) and long-term trend effects (1.36 times, 95% CI: 1.33–1.39, *p* < 0.001), compared to older youth (1.09 and 1.21 times) (*p* < 0.001) (Figure 9c,d). Significant seasonal patterns occurred for ages 13–17 years comprising an increase of 1.49 times (95% CI: 1.47–0.51) in the second quarter (Figure 9e) and decrease of 0.92 times (95% CI: 0.90–0.93) in the fourth quarter (Figure 9g). The concurrent availability of COVID-19 vaccines stimulated HPV administration among younger youth (1.16 times, 95% CI: 1.08–1.25, *p* < 0.01), but had a modest competitive effect on older youth (0.98 times, 95% CI: 0.97–0.99, *p* < 0.01) (Figure 9i).

#### 3.6.4. Initiation Rate Modeling for Meningococcal (MCV4) Vaccine

**Figure 10:**
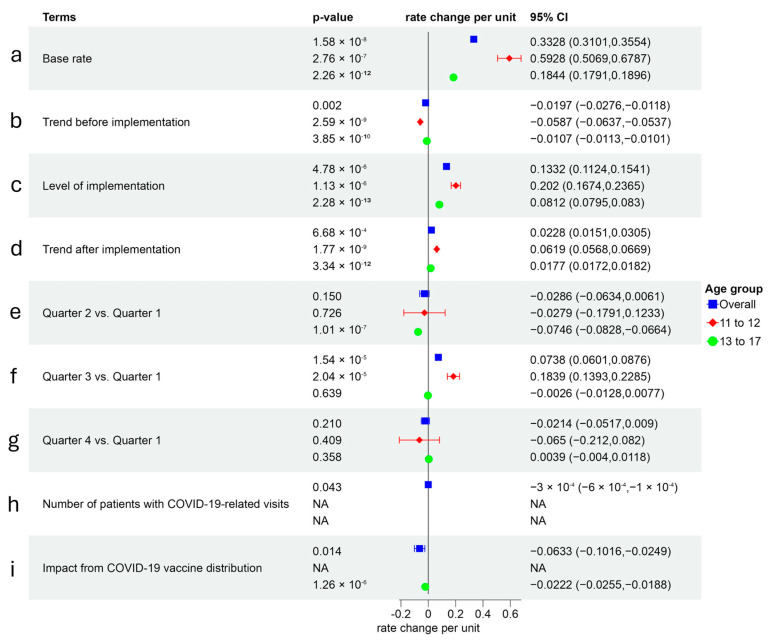
Initiation rate modeling for meningococcal (MCV4) vaccine.

MCV4 vaccine initiation rates were also influenced by trends, AVP-IT implementation, seasonality, and COVID-19 factors (Figure 10). A prior (pre-implementation) downward trend reduced MCV4 initiation proportions by 0.02 (95% CI: −0.03–0.01, *p* < 0.01) (Figure 10b), while AVP-IT implementation was associated with increased rates by 0.13 (95% CI: 0.11–0.15, *p* < 0.001) (Figure 10c). The long-term implementation trend also yielded a smaller but significant increase (0.02, 95% CI: 0.02–0.03, *p* < 0.001) (Figure 10d). However, the distribution of COVID-19 vaccines negatively impacted MCV4 initiation rates, reducing vaccination rates by 0.06 (95% CI: −0.10–−0.03, *p* < 0.01) (Figure 10i). Younger youth exhibited stronger responses to AVP-IT implementation (0.20, 95% CI: 0.17–0.24, *p* < 0.001) (Figure 10c) and long-term trends (0.06, 95% CI: 0.06–0.07, *p* < 0.001) compared to older youth (0.08 and 0.02, respectively, *p* < 0.001) (Figure 10d). Unlike HPV vaccines, MCV4 initiation rates were less influenced by seasonality among older youth.

#### 3.6.5. Initiation Rate Modeling for Tetanus, Diphtheria, and Acellular Pertussis (Tdap) Vaccines

**Figure 11:**
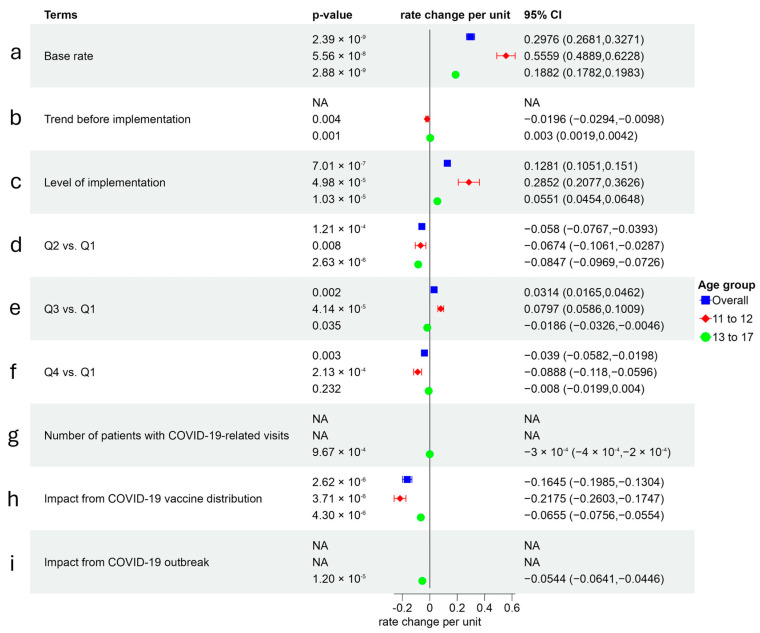
Initiation rate modeling for tetanus, diphtheria, and acellular pertussis (Tdap) vaccines.

For Tdap vaccines, the primary effect was observed in an immediate association of AVP-IT implementation (Figure 11). AVP-IT was associated with increased initiation rates by 0.13 (95% CI: 0.11–0.15, *p* < 0.001) overall, with stronger effects among younger (0.29, 95% CI: 0.21–0.36, *p* < 0.001) compared to older (0.06, 95% CI: 0.05–0.07, *p* < 0.001) youth (Figure 11c). Baseline trends showed an opposite direction for age groups: younger youth experienced a declining trend (−0.02, 95% CI: −0.03–−0.01, *p* < 0.05), whereas older youth exhibited a slight increase (0.003, 95% CI: 0.002–0.004, *p* < 0.01) (Figure 11b). The COVID-19 pandemic further suppressed Tdap vaccination rates through varying mechanisms, including COVID-19 outbreaks and competing vaccine distributions, which had stronger impacts on younger youth (Figure 11i).

### 3.7. Recommended Enhancements of the AVP-IT

This study has supported earlier reported usability results that highlighted facilitators and barriers for AVP-IT adaptation to the safety-net clinic context that inform future enhancements of the AVP-IT [46] (Table 5).

## 4. Discussion

This study demonstrated that an online adaptation of the evidence-based Adolescent Vaccination Program (AVP)—the AVP Implementation Tool (AVP-IT)—was feasible to provide decision support in safety-net clinics for the purpose of increasing HPV vaccination and improving primary healthcare for the medically underserved. ITS analysis, after adjusting for confounders, demonstrated positive associations of the AVP-IT with HPV, MCV, and Tdap vaccination rates, including both immediate stimulation and favorable long-term trends. The AVP-IT implementation was intended to improve HPV vaccination rates, but an association with increased MCV4 and Tdap vaccination volumes was also observed. This was not observed in previous AVP studies [33,36]. This finding may be beneficial to mitigate hesitancy toward the use of vaccination within the U.S. The AVP-IT was associated with a boost in vaccination rates longitudinally. This may have implications for long-term maintenance and sustainability of evidence-based strategies to increase HPV, MCV4, and Tdap vaccinations.

Consistent with previous age-related gaps, youth of ages 11–12 years had stronger natural declines in vaccination rates compared to youth of ages 13–17 years, possibly indicating greater vaccine hesitancy among parents of younger-age youth [70,71]. Conversely, the AVP-IT intervention was associated with a stronger immediate increase and stronger long-term trajectory among the younger cohort (11–12 years) compared to the older cohort (13–17 years) for all three vaccines. The AVP may be particularly salient for the parents of younger adolescents. The age-related ‘rate gap’ narrowed after AVP-IT implementation, suggesting that the AVP-IT may help to reduce age-related disparities over time. Clinic leaders provided supporting anecdotal information that parents believed that the HPV vaccine is only effective within a specific time range up to 13 years of age, and if they miss this window, then they just ‘let it go.’ To capitalize on the responsiveness of parents of younger adolescents in this study, and in accordance with numerous guidelines, the AVP-IT will be modified to promote HPV initiation at age 9 in CME and CNE material, provider prompt algorithms, parent message reminder triggers, and the parent HPVcancerFree app [15,16,17,18,19,20,21]. The AVP-IT will be further enhanced by including tailored messaging and reminders for parents with youth aged 13–17 years to overcome the misperception that the ‘vaccination window’ closes after age 13.

The study demonstrated context-specific effects of the COVID-19 pandemic on vaccination rates, mostly exerting a strong negative association. The pandemic led to increased competition for limited medical resources, with health systems prioritizing emergency care and COVID-19 vaccination efforts over routine immunizations. Incorporating public health emergencies into design and timing of routine vaccination strategies may help to mitigate future disruptions. The concurrent availability of COVID-19 vaccines was associated with greater impact on HPV vaccine administration among younger youth compared to older youth, contributing to an age discrepancy. However, overall, there appeared to be a positive synergistic effect from COVID-19 vaccine distribution, which was associated with improved HPV vaccination rates.

The original AVP was a multi-component program delivered in person in clinic settings and demonstrated to successfully increase HPV vaccination [33,36]. This was achieved through the adoption and implementation of evidence-based strategies comprising assessment and feedback to providers on vaccination successes and missed opportunities, prompts to providers that provided real-time alerts about vaccine-eligible youth, reminders to parents that their youth were eligible for vaccines, presumptive bundled messages from HCPs to parents within the clinic visit, and parent education to mitigate misinformation and hesitancy [27,28,29]. The conventional ‘in-person’ approach used in the AVP is consistent with training and technical assistance that has overcome individual and organizational barriers to HPV vaccination, but this can be resource-intensive [70]. The notion of online decision support to provide for more efficient, consistent, and scalable ‘virtual’ dissemination and reach across safety-net clinics with minimum resource overhead is therefore attractive.

The AVP-IT functioned as designed with no reported ‘bugs’ or technical problems that would have prevented the creation and download of the tailored Action Plan. It remained accessible to clinic staff throughout the trial and no negative or harmful effects were observed as a result of completing the HPV vaccination strategies. Despite this, there were caveats to the AVP-IT implementation. For example, this study highlighted important facilitators and barriers to the successful deployment of the AVP-IT [44,72,73,74,75,76,77,78,79]. The *a priori* tenet that the AVP-IT decision support could be ‘stand-alone’, requiring little-to-no support from research staff outside the clinic, was not fully realized. Champions completed the AVP-IT Action Plan Wizard and obtained their tailored Action Plans without any trouble. However, little progress in strategy implementation was reported during the first months. Despite clinic leadership support and a champion orientation webinar, there was initial inertia. This was overcome with an extra level of technical support via monthly virtual review meetings with research staff to review the champion checklist. This technical support appeared necessary even when the online decision support and clinic leadership support were in place. It was apparent that ‘if you build it, they won’t necessarily come.’ Not all the evidence-based strategies promoted by the AVP were fully implemented, while, conversely, implementation of a strategy (e.g., assessment and feedback) was not necessarily dependent on completion of all champion tasks.

Support exists in the literature for the efficacy of the HPV vaccination strategies promoted in the AVP-IT, but not all strategies were implemented with equal fidelity. For example, prompts to providers that vaccine-eligible youth are in the clinic is an important strategy to lower missed opportunities to vaccinate, and is one of the evidence-based strategies implemented through the AVP-IT. Implementation of provider prompts moved in a positive direction, with one clinic fully implementing this strategy in accordance with AVP-IT recommendations. This result was modest. In the analysis, the AVP-IT was conceptualized as an integrated comprehensive intervention targeting healthcare providers, vaccination champions, and parents simultaneously, rather than as separable, decomposable, components consisting of clinic-level task adherence metrics or consecutive strategy rollouts. The primary research goal was to evaluate whether population-level vaccination initiation rates changed following AVP-IT implementation, with the AVP-IT considered as a single intervention over time. Future studies could investigate the mechanisms of effect of different strategies like provider prompts in the context of FQHCs and safety-net clinics, or assess between-clinic differences in vaccination rates based on the degree of different strategy implementation. This could incorporate modeling of dose–response relationships based on partial fidelity measures (described in Table 2) and take into account mediation or effect-modification analyses of implementation intensity and component-specific effects. Such a ‘reductionist’ approach could yield more understanding of the mechanisms of individual strategies.

Provider prompts, parent reminders, and assessment and feedback reports require valid and reliable vaccination data. Providing these is a fundamental objective for EHR optimization. The study did not include EHR optimization, but the findings of this study suggest an optimization road map for the AVP-IT. This will include assignment of a dedicated Quality Improvement Advisor from a Biomedical Informatics Center for Quality Health IT Improvement to each participating clinic. This advisor will collaborate with the clinic’s designated champion or IT personnel to: (1) optimize clinic workflows, (2) integrate EHR-based tools, and (3) provide staff training to ensure effective EHR utilization. Provider prompts will be developed and tested prior to ‘going live’, which triggers schedule logic using age and dose data. Recommended provider prompt logic for eligibility for HPV vaccine initiation is exemplified as follows: ‘IF patient has not received a dose of the HPV vaccine AND patient is female 9–26 years of age OR male 9–21 years of age THEN patient is eligible for HPV#1.’ Minimum value rule sets will be established that set provider response criteria (e.g., 80%) to ensure minimal missed opportunities through comparison of prompt firing rates against vaccination success. Parent reminders will be established subsequent to internal quality control to ensure patient contact information is up to date in the EHR. Patient reminder systems will be developed for patients eligible for HPV vaccine by age, and also for patients due for a second or third dose based on ACIP schedule. Recommended parent reminder logic for eligibility for HPV vaccine initiation is exemplified as follows: ‘IF patient has not received a dose of the HPV vaccine AND patient is female 9–26 years of age OR male 9–21 years of age THEN patient is eligible for HPV#1.’ Setting up automated reminder systems and registries can be challenging for smaller clinic systems. Establishing interoperability with state registries or, at least, access to data in registries can facilitate this. Minimum value rule sets will be established that set reminder output to all eligible youth against vaccination to meet a criterion of 80%.

Numerous countries have engaged in implementing HPV vaccination strategies. Effective rate increases of approximately 80% have been reported as a result of programs that integrate school-based delivery, national immunization registries, and digital reminder systems for parents [80,81]. Australia has achieved over 80% coverage for adolescent girls by triangulating the national Australian Immunization Register (AIR) record of doses with an embedded automated reminder system that provides alerts about missed doses, and a school-based vaccination program with SMS reminders to parents [82]. In the United Kingdom, rates of over 85% have been achieved through the application of school-based programs that include reminders for parents to return consent forms, digital decision support for parents, and digitally mediated ‘catch-up’ campaigns via emails, texts, and apps for ‘missed opportunity’ youth [81]. Canadian HPV vaccination rates range from 70 to 90% as a result of a school-based approach with concurrent online digital decision support for parents that addresses vaccination concerns, and Denmark has demonstrated vaccination increases using an integrated EHR to identify eligible youth and trigger alerts for vaccination in a ‘prospective reminder policy’ [81,83]. The AVP-IT is operating in Texas, which does not mandate the HPV vaccine, lacks school-based programming [84], and often lacks integrated EHRs in community clinics. However, optimizing EHRs in clinics for bidirectional data exchange with the state (IMM-Trac) registry and applying digital reminders and decision support for parents are indicated, given the international findings [80,81]. AI-driven decision support chatbots have demonstrated improvement in HPV-related literacy and increased vaccine uptake among parents in China, representing ‘next-gen’ opportunities for HPV vaccine promotion [85].

The results of this pilot study need to be interpreted in the light of several limitations. The extended follow-up of 33 months with a larger sample of clinics enabled a more robust assessment of the implementation and impact of the AVP-IT on HPV vaccination rates than was achieved in the previously published pilot [46]. However, the observational window may still have been insufficient to fully capture long-term trends, particularly given the gradual effects of AVP-IT strategies. Data were collected within 16 quarters, fewer than the recommended 24 quarters for trend analysis [65]. This may have led to reduced statistical power, contributing to discrepancies between ITS-predicted rates and raw quarterly means. Vaccination initiation rates are subject to inherent short-term fluctuations, which were further exacerbated by external disruptions such as the COVID-19 pandemic, and patient migration may have introduced bias, as youth moving between locations and clinics could have affected both vaccination administration and data stability. Further, the EHR was not optimized for interoperability to capture vaccination records outside participating clinics, resulting in incomplete data entries that may have undermined the accuracy of results. Despite these limitations, ITS remains the most appropriate analytic framework for this study, as it is specifically designed to characterize temporal trends and intervention effects in the absence of concurrent control groups [60].

The promotion of bundled messaging in the AVP-IT may have contributed to MCV4 and Tdap vaccination rate increases, but it is difficult to attribute causality to the AVP-IT without considering the possibility of broader quality initiatives and community influences such as school entry requirements. Related financial considerations and administrative decision making related to vaccine distribution (where MCV4/Tdap may be considered more cost-effective compared to HPV vaccines) have not been included in this study, but could be contributing factors. Expanding the inquiry to include a comparison of county or state vaccination rates, fiscal strategies, and other spillover mechanisms may assist with gaining greater insight into the underlying contributing factors.

The strategies promoted in this study are evidence-based, with broad-based effectiveness. However, caution is advised when interpreting the generalizability of the study findings to other FQHCs and safety-net clinics beyond Houston. This study was limited to a single urban safety-net clinic network, with limited patient demographics and insurance and self-pay coverage, subject to COVID-19 pandemic influence, and operated without an optimized EHR. Irrespective of this, the AVP-IT is among the first decision support tools designed to enable pediatric and community clinics to autonomously implement evidence-based strategies to increase HPV vaccination rates. The utility of the use of an Action Plan was demonstrated, with positive movement toward strategy adoption from ‘partial’ to ‘full’ implementation. This work supports ongoing initiatives from federal (NCI, CDC), non-profit (ACS), state (CPRIT, HPV Coalition of Texas), and local organizations (MDA, UTHealth) to mitigate individual (attitudinal)- and organizational (infrastructure)-level barriers to HPV vaccination. Grant mechanisms provide resources that enable remuneration to offset costs for initiatives like the AVP-IT to embed evidence-based practices. This is an important policy consideration, particularly for FQHCs. More rigorous future RCTs are recommended to demonstrate the relative efficacy of this online decision support strategy when compared with usual practice. A controlled ITS or cluster-randomized stepped-wedge trial is indicated subsequent to EHR optimization (including prompts, reminders, and dashboards) to promote maximal impact. This study did not report on vaccination completion rates to provide a more comprehensive understanding of the AVP-IT in approximating national goals. This should be considered for future studies. Cost-effectiveness evaluation is also recommended to facilitate any planned AVP-IT scale-up.

## 5. Conclusions

The AVP-IT and the provision of a stepped, tailored, clinic-based Action Plan appear feasible for use in safety-net clinics, but for optimal efficacy, their use is recommended in conjunction with technical assistance consistent with the original in-person AVP program. Clinic-based AVP-IT use was associated with increased HPV, MCV, and Tdap vaccination rates over a 33-month period even though not all evidence-based strategies were fully implemented. The AVP-IT was not used by clinical staff as a ‘standalone’ intervention as originally designed. Even with leadership support, its use required mediation with technical assistance from the research team. Leadership support, technical assistance for AVP champions and EHR optimization are all strongly recommended as foundational for the AVP-IT evidence-based strategies to be fully implemented. The results need to be interpreted in the context of study limitations that include limited clinic sample size and study duration of 16 quarters, and no comparison condition to limit threats to internal validity. Despite this, the study supports the use of the AVP-IT to help facilitate the implementation of evidence-based strategies to increase vaccination and healthcare for the underinsured and underserved in a safety-net clinic context.

## Figures and Tables

**Table 1:** Youth sample demographics.

Features	Pre-Implementation (N = 4351) ^1^ n (%)	Post-Implementation (N = 3943) ^1^ n (%)	*p*-Value ^2^
Age (mean ± SD) ^3^	14.03 ± 1.7	13.87 ± 1.69	<0.001
Number of clinic visits (mean ± SD)	1.59 ± 1.1	1.60 ± 1.08	0.75
Gender			
Female	2131 (48.9)	1893 (48.0)	0.59
Male	2218 (50.9)	2049 (51.9)	
Undifferentiated	2 (0.02)	1 (0.01)	
Race			
African American	319 (7.3)	280 (7.1)	0.038
Asian	388 (8.9)	288 (7.3)	
Hispanic	605 (13.9)	514 (13.0)	
White	97 (2.2)	109 (2.8)	
Others ^4^	14 (0.3)	14 (0.4)	
Patient Declined/Unknown	2928 (67.3)	2738 (69.4)	
Insurance status ^5^			
Commercial FFS	37 (0.9)	39 (0.9)	<0.001
Medicaid FFS	24 (0.6)	58 (1.5)	
Self-Pay	1899 (43.6)	3411 (86.5)	
Other	2359 (54.2)	350 (8.9)	
COVID-19 HRSA Uninsured Testing and Treatment Fund	2199	101	
Others	160	249	
Missing	32 (0.7)	85 (2.2)	

^1^ Youth with available demographic data included, respectively, 4351 and 3943 before and after AVP-IT implementation. This included 629 youth who had multiple visits before and after AVP-IT implementation. ^2^ Two-sample *t*-tests and chi-square tests were performed to detect the difference in distribution for continuous and categorical variables, respectively. ^3^ The shift in mean age over time could be due to geographic relocation, patient healthcare preferences, broader COVID-19-related policy-related changes, and/or healthcare transition of older youth out of pediatric care. ^4^ Other races include American Indian, Native Hawaiian, and Other Pacific Islander. ^5^ The distribution of the COVID-19 testing fund caused an imbalanced distribution. If COVID-19 funding is not included the resulting p-value for insurance status is 0.0136.

**Table 2:** AVP champion implementation task fidelity for strategies by clinic site *.

Strategies and Related Tasks from the Champion Checklist		Clinic Sites				Adh. (%)
		1	2	3	4	
CONTINUING EDUCATION (CME/CNE)						
□	Print the AVP CME/CNE flyer & notify clinic staff at clinic meetings and/or one-on-one	y	y	y	y	100
□	Distribute the AVP CE flyer to clinic staff		y	y		50
□	Hang the AVP CE flyer in areas of your clinic that are highly visible to staff	y		y	y	75
□	Send an email to clinic staff with information about the AVP CE		y			25
□	Set up & inform staff of the timeline for completion (30 days)		y	y	y	75
□	View the video presentation (self and staff)	y	y	y	y	100
□	Have staff that have completed the CE sign the AVP CE Tracking/Sign-Off Form		y	y		50
□	Provide the CE to new staff during their onboarding	y	y	y	y	100
□	Collect and file copies of CME/CNE certificates		y			25
	Adherence (%)	44	89	78	56	
PROVIDER PROMPTS (For non-optimized EHR) *						
□	Construct a manual report of eligible patients due for HPV vaccination	y	y	y	y	100
□	Assign a staff member to place notes in medical records of eligible patients	-	y	y	-	50
	Adherence (%)	50	100	100	50	
PARENT REMINDERS						
□	Review parent reminder best practices with clinic staff	y	y	y	y	100
□	Review youth contact information to ensure it is up to date (reminder quality control)	y	y	-	y	75
□	Use the AVP parent reminder logic to determine vaccine eligibility	y	y	y	y	100
□	Construct a manual report of eligible youth due for the first dose of the HPV vaccine	y	-	-	-	25
□	Construct a manual report of eligible youth due for follow-up doses of the HPV vacc.	-	-	-	y	25
□	Assign a staff member to send reminder emails or phone calls to eligible youth	y	y	-	-	50
□	Provide parents with written reminders for follow-up doses—After dose administration and logging, MAs print an ImmTrac recall letter of due date for follow-up dose/s	y	y	-	-	50
□	MAs schedule follow-up dose appointments before the patient leaves the clinic	-	y	y	y	75
□	MA/front desk staff makes a note in the calendar when the patient is due for their next dose	-	y	y	y	75
	Adherence (%)	67	78	44	67	
PARENT EDUCATION—HPVcancerFree app (HPVCF))						
□	Distribute the HPVCF flyer, and/or send an email to clinic staff, with information about the HPVCF; notify them at clinic meetings	-	y	y	y	75
□	Hang the HPVCF flyer in the clinic	-	y	y	y	75
□	Hang Spanish-language posters in the clinic	y	y	y	y	100
□	Provide clinic staff with copies of the HPVCF flyer (including Spanish resources/materials) and have them distribute these to eligible parents	-	y	y	-	50
□	Review instructions for downloading the app with clinic staff					
	Adherence (%)	25	80	80	60	

* AVP champion selection and onboarding are not presented because these tasks were accomplished a priori. Assessment and feedback are not presented because implementation was pending EHR optimization. Provider prompts and parent reminders are reported for non-optimized EHR because tasks to team up with the EHR provider to configure provider prompts and reminders were beyond scope.

**Table 3:** AVP-IT strategy implementation outcomes in the participating clinics before (n = 5) and after (n = 4) AVP-IT implementation.

Evidence-Based Strategies and Action Plan Wizard Items ^1^		Pre-Implementation (n = 5)					Post-Implementation (n = 4)				
		Yes ^2^ (n)	No ^2^ (n)	Status (n) ^3^			Yes ^2^ (n)	No ^2^ (n)	Status (n) ^3^		
				Pend	Part	Full			Pend	Part	Full
AVP Champion	Is there an immunization champion in your clinic?	3	2	2	1	2	3	1	1	-	3
	IF YES, does your champion devote time to HPV vaccination quality improvement?	2	1				3				
Assessment and Feedback	In your clinic, are the HCPs given regular feedback (at least quarterly) on their HPV vaccination rates?	2	3	3	1	1	4		-	2	2
	IF YES, does the feedback contain provider- and clinic-level data on all adolescent vaccinations, including comparisons between providers in your clinic and with national or clinic goals?	1	1				2	2			
Continuing Education	In your clinic, are providers receiving continuing education on HPV vaccination?	4	1	1	4	-	3	1	1	1	2
	IF YES, are your HCPs receiving CME or CNE with ethics credit that covers the latest training on HPV, HPV vaccination, evidence-based strategies, and best practices to navigate patient resistance?		4				2	1			
Provider Prompts	In your clinic, do your HCPs receive prompts (in the EHR or otherwise) that a patient is eligible for the HPV vaccine?	1	4	4	-	1	1	3	3	-	1
	IF YES, do the reminders identify patients who are both due and overdue for any dose of the HPV vaccine?	1					1				
Parent Reminders	In your clinic, do you send reminders (e-mail/text/phone/mail) to parents when their child is eligible for HPV vaccinations?	2	3	3	-	2	3	1	1	-	3
	IF YES, do the reminders identify patients who are both due and overdue for any dose of the HPV vaccine?	2					3				
Parent Education	In your clinic, do you provide educational material on the HPV vaccine to your patients?	4	1	1	4	-	4		-	-	4
	IF YES, do you provide any self-tailored phone-based Apps to raise their awareness about the importance and safety of HPV vaccination and also address myths and barriers surrounding HPV vaccination?		4				4				

^1^ Action Plan Wizard items on the AVP-IT website; ^2^ The grayed region indicates the number of champions responding to each AVP-IT wizard survey item. ^3^ Status refers to the fidelity of implementation. Pending (Pend) = does not perform; Partially (Part) = HPV evidence-based strategy is being performed but not in accordance with the AVP Action Plan criteria; Fully (Full) = implemented in accordance with the AVP Action Plan.

**Table 4:** Summary of modeling elements.

Vaccine	If Log-Transferred	Population Group	Trend Before Implementation	Level of Implementation	Trend After Implementation	Quarter	Number of COVID-19-Related Visits	Impact from COVID-19 Vaccine Distribution	Impact from COVID-19 Outbreak
HPV	y	Overall	y	y	y	y	y		
		11 to 12	y	y	y	y	y	y	
		13 to 17	y	y	y	y	y	y	
MCV4		Overall	y	y	y	y	y	y	
		11 to 12	y	y	y	y			
		13 to 17	y	y	y	y		y	
Tdap		Overall		y		y		y	
		11 to 12	y	y		y		y	
		13 to 17	y	y		y	y	y	y

**Table 5:** Recommended enhancements to strategies for AVP-IT implementation in safety-net clinics.

AVP Strategy	Factor	Description of Proposed AVP Enhancements
AVP Clinic Champion Training	Leadership	Regular status updates and ongoing collaboration, increased familiarity with AVP [34].
	Staff turnover	Champion identification and onboarding protocols.
	Training	Well-defined expectations, webinars, leadership (and lead champion) oversight.
	Decision aids	Task checklists & monitoring calls were introduced to complement the existing online AVP-IT & tailored Action Plan. An additional data management guideline is proposed.
Provider assessment & feedback	EHR data	Institution of data extraction and management protocols & EHR optimization & interoperability. A non-optimized EHR and limited interoperability cause outcome misclassification. A linkage audit is proposed with the state registry (ImmTrac) as a data quality check.
	Leadership	Enhancement of clinic practice to improve record keeping and tracking of vaccinations.
	Decision aids	Existing template for report generation.
Provider CME	Accessibility	Update and enhancement of CME accessibility via the online AVP-IT website modification and additional ethics credit.
	Updates	CME updates with recommendations (ages 9–12) and social media influence on hesitancy.
Provider prompts	EHR algorithm	Real-time HPV provider cues in EHR platforms (e.g., NextGen, Practice Fusion). See also the note on EHR optimization regarding assessment and feedback above.
Patient reminders	Tailored messaging	Upgraded reminder protocols informed by AVP algorithms and using the existing EHR and Imm-Track data system. Theory- and empirically based messaging (email and text). See also the note on EHR optimization regarding assessment and feedback above.
HPVcancerFree patient education app.	Targeting younger ages	Enhanced content & messaging to include males and females (9–17 yrs.) reflecting AAP and ACS recs [68] for HPV vaccination at age 9 because earlier initiation is associated with increased parental acceptance and rates of series completion by age 13 [69].
	Literacy	Spanish-language CDC materials were introduced to clinic leadership within the current study but AVP HPVcancerFree app modification for language and literacy is indicated.
	Culture	Adaptation of patient education material to fit the cultural needs of Hispanic patients.

## Data Availability

The deidentified aggregated dataset presented in this study are available on request from the corresponding author. The data are not publicly available due to security restrictions.

## Supplementary Materials

The following supporting information can be downloaded at: https://www.mdpi.com/article/10.3390/healthcare14040519/s1, Table S1: Statistics of Breusch-Godfrey test; Table S2: ITS modelling parameters of log-transferred undue HPV initiation rates; Table S3: ITS modelling parameters of undue MCV4 initiation rates; Table S4: ITS modelling parameters of undue Tdap initiation rates; Figure S1: Undue HPV vaccination volume and eligible patients per quarter; Figure S2: Undue MCV4 vaccination volume and eligible patients per quarter; Figure S3: Undue Tdap vaccination volume and eligible patients per quarter; Figure S4: Autocorrelation of vaccination volume; Figure S5: Model diagnostic of HPV vaccination modelling; Figure S6: Model diagnostic of MCV4 vaccination modelling; Figure S7: Model diagnostic of Tdap vaccination modelling.

## Abbreviations

The following abbreviations are used in this manuscript:

AVP	Adolescent vaccination program
AVP-IT	Adolescent vaccination program—implementation tool
CME	Continuing medical education
CNE	Continuing nursing education
COVID-19	Coronavirus disease of 2019
CVX	Code standardized to a particular vaccine product administered
EBS	Evidence-based strategy
EHR	Electronic health record
FQHC	Federally funded health clinic
HCP	Healthcare provider
HPV	Human papillomavirus
MA	Medical assistant
MCV4	Meningococcal conjugate vaccine
Tdap	Diphtheria, tetanus, and acellular pertussis vaccine
