# Supplementary material for: Online Decision Support for Implementing Evidence-Based HPV Vaccination Strategies in Texas Safety-Net Pediatric Clinics: Impact on HPV, MCV, and Tdap Initiation
Source: Healthcare (Basel). 2026 Feb 18;14(4):519. doi: 10.3390/healthcare14040519 (PMC12940289; doi:10.3390/healthcare14040519)

Online Decision Support to Implement Evidence-Based HPV Vaccination Strategies in Texas Safety-Net Pediatric Clinics: Impact on HPV, MCV, and Tdap Initiation.

Supplementary Materials

Supplementary Table S1. Statistics of Breusch-Godfrey test

Vaccine	Highest order autocorrelation tested	Overall population		11 to 12 years old		13 to 17 years old	
		coefficient	p-value	coefficient	p-value	coefficient	p-value
HPV	1st	1.8211	0.1722	0.0009	0.9764	6.4093	0.0114
	4th	10.4560	0.0334	11.9180	0.0180	14.3340	0.0063
MCV4	1st	0.5201	0.4708	0.1912	0.6619	6.8369	0.0089
	4th	10.7040	0.0301	7.5883	0.1079	10.7350	0.0297
Tdap	1st	1.9924	0.1581	0.9040	0.3417	0.6729	0.4121
	4th	8.8919	0.0639	7.3091	0.1204	8.1280	0.0870

This table documents Breusch–Godfrey test statistics assessing serial correlation at the first order and at the fourth order, with the latter jointly testing autocorrelations from lags 1 through 4. The results provide supplementary evidence for evaluating whether first- or fourth-order autocorrelation should be considered in the modeling framework, but they shouldn't be served as the sole determinant, given that the fourth-order test simultaneously evaluates lower-order lags. Accordingly, decisions regarding the appropriate autocorrelation structure were informed by both the Breusch–Godfrey test results (Table S1) and the autocorrelation diagnostics presented in Supplementary Figure S4.

Supplementary Table S2. ITS modelling parameters of log-transferred undue HPV initiation rates

population	term	coef	s.e.	t-value	p-value	s.e. of residuals
overall	Intercept	-1.3384	0.1398	-9.5751	1.17E-05	0.2317
	t1	-0.207	0.0201	-10.2805	6.90E-06	
	AVP_IT_implementation	0.5952	0.1131	5.2622	7.62E-04	
	t2	0.2108	0.0291	7.2478	8.82E-05	
	quarter2	0.1803	0.1438	1.2539	0.2453	
	quarter3	0.5239	0.1658	3.1607	0.0134	
	quarter4	-0.0767	0.1458	-0.5259	0.6132	
	n_covid	-0.007	0.0013	-5.5649	5.32E-04	
11 to 12	Intercept	-0.2431	0.0397	-6.128	4.78E-04	0..2785
	t1	-0.3303	0.0078	-42.3145	1.07E-09	
	AVP_IT_implementation	0.8569	0.0335	25.5758	3.57E-08	
	t2	0.3079	0.0103	30.0332	1.17E-08	
	quarter2	-0.253	0.0340	-7.446	1.44E-04	
	quarter3	0.1024	0.0419	2.4413	0.0447	
	quarter4	-0.157	0.0269	-5.8447	6.34E-04	

	n_covid	-0.0028	0.0001	-24.9727	4.21E-08	
	t_covid_vaccine	0.1522	0.0379	4.0148	0.0051	
13 to 17	Intercept	-2.2802	0.0035	-651.87	5.28E-18	0.3272
	t1	-0.1228	0.0007	-172.2178	5.87E-14	
	AVP_IT_implementation	0.0901	0.0025	35.4891	3.66E-09	
	t2	0.19	0.0004	434.1949	9.08E-17	
	quarter2	0.4013	0.0065	62.082	7.39E-11	
	quarter3	0.6444	0.0049	130.9364	4.00E-13	
	quarter4	-0.0871	0.0066	-13.2581	3.25E-06	
	n_covid	-0.0118	4.89E-05	-241.9327	5.44E-15	
	t_covid_vaccine	-0.0234	0.0049	-4.7388	0.0021	

Annotation of terms: intercept: base rate, t1: trend before implementation, AVP_IT_implementation: level of implementation, t2: trend after implementation, quarter 2: Quarter 2 vs. Quarter 1, quarter 3: Quarter 3 vs. Quarter 1, quarter 4: Quarter 4 vs. Quarter 1, n_covid2: number of patients with COVID-related vaccine distribution, t_covid_vaccine: impact from COVID vaccine distribution, t_covid_outbreat: impact from COVID outbreak.

Supplementary Table S3. ITS modelling parameters of undue MCV4 initiation rates

population	term	coef	s.e.	t-value	p-value	s.e. of residuals
overall	Intercept	0.3328	0.0116	28.767	1.58E-08	0.0204
	t1	-0.0197	0.004	-4.8822	0.0018	
	AVP_IT_implementation	0.1332	0.0106	12.5207	4.78E-06	
	t2	0.0228	0.0039	5.7942	6.68E-04	
	quarter2	-0.0286	0.0177	-1.6144	0.1505	
	quarter3	0.0738	0.007	10.5146	1.54E-05	
	quarter4	-0.0214	0.0155	-1.3788	0.2104	
	n_covid	-0.0003	0.0001	-2.4697	0.0429	
	t_covid_vaccine	-0.0633	0.0196	-3.2351	0.0143	
11 to 12	Intercept	0.5928	0.0438	13.5278	2.76E-07	0.0636
	t1	-0.0587	0.0025	-23.0513	2.59E-09	
	AVP_IT_implementation	0.202	0.0176	11.4647	1.13E-06	
	t2	0.0619	0.0026	24.0557	1.77E-09	
	quarter2	-0.0279	0.0771	-0.3612	0.7263	
	quarter3	0.1839	0.0228	8.0834	2.04E-05	
	quarter4	-0.065	0.075	-0.8668	0.4086	
13 to 17	Intercept	0.1844	0.0027	68.6468	2.26E-12	0.0124
	t1	-0.0107	0.0003	-36.0337	3.85E-10	
	AVP_IT_implementation	0.0812	0.0009	91.4611	2.28E-13	
	t2	0.0177	0.0003	65.3536	3.34E-12	
	quarter2	-0.0746	0.0042	-17.8197	1.01E-07	
	quarter3	-0.0026	0.0052	-0.4869	0.6394	
	quarter4	0.0039	0.004	0.9756	0.3578	
	t_covid_vaccine	-0.0222	0.0017	-12.8659	1.26E-06	

Same term annotations as Supplementary Table S2.

Supplementary Table S4. ITS modelling parameters of undue Tdap initiation rates

population	term	coef	s.e.	t-value	p-value	s.e. of residuals
overall	Intercept	0.2976	0.015	19.7836	2.39E-09	0.0282
	AVP_IT_implementation	0.1281	0.0117	10.9268	7.01E-07	
	quarter2	-0.058	0.0096	-6.0681	1.21E-04	
	quarter3	0.0314	0.0076	4.1398	0.0020	
	quarter4	-0.039	0.0098	-3.9774	0.0026	
	t_covid_vaccine	-0.1645	0.0174	-9.4658	2.62E-06	
11 to 12	Intercept	0.5559	0.0342	16.2696	5.56E-08	0.0524
	t1	-0.0196	0.0050	-3.9099	0.0036	
	AVP_IT_implementation	0.2852	0.0395	7.2192	4.98E-05	
	quarter2	-0.0674	0.0198	-3.4093	0.0078	
	quarter3	0.0797	0.0108	7.3914	4.14E-05	
	quarter4	-0.0888	0.0149	-5.9589	2.13E-04	
	t_covid_vaccine	-0.2175	0.0218	-9.9578	3.71E-06	
13 to 17	Intercept	0.1882	0.0051	36.7307	2.88E-09	0.0183
	t1	0.003	0.0006	5.0490	0.0015	
	AVP_IT_implementation	0.0551	0.0049	11.1573	1.03E-05	
	quarter2	-0.0847	0.0062	-13.6767	2.63E-06	
	quarter3	-0.0186	0.0071	-2.6089	0.0350	
	quarter4	-0.008	0.0061	-1.3099	0.2316	
	n_covid	-0.0003	0.0001	-5.4391	9.67E-04	
	t_covid_vaccine	-0.0655	0.0051	-12.7195	4.30E-06	
	t_covid_outbreak	-0.0544	0.0050	-10.9055	1.20E-05	

Same term annotations as Supplementary Table S2.

Supplementary Figure S1. Undue HPV vaccination volume and eligible patients per quarter.

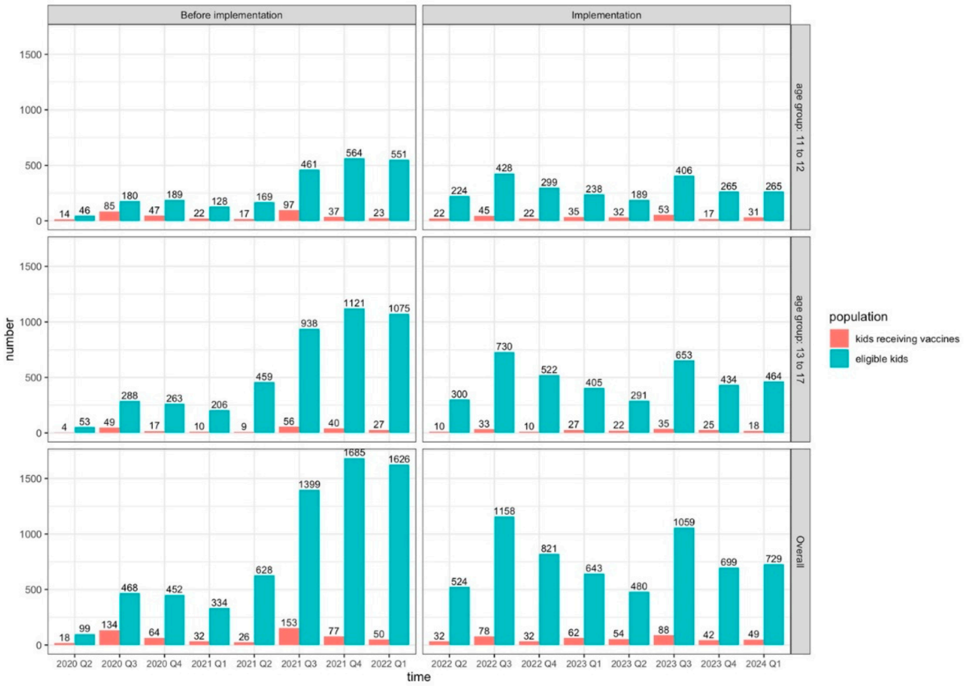

Supplementary Figure S2. Undue MCV4 vaccination volume and eligible patients per quarter.

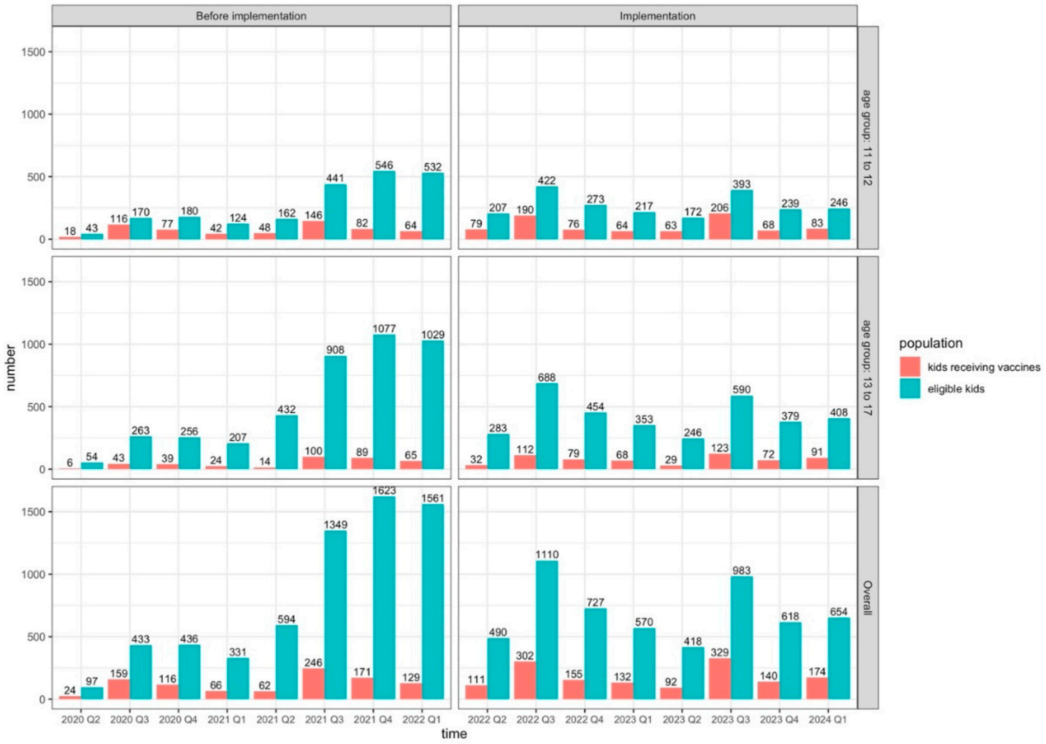

Supplementary Figure S3. Undue Tdap vaccination volume and eligible patients per quarter.

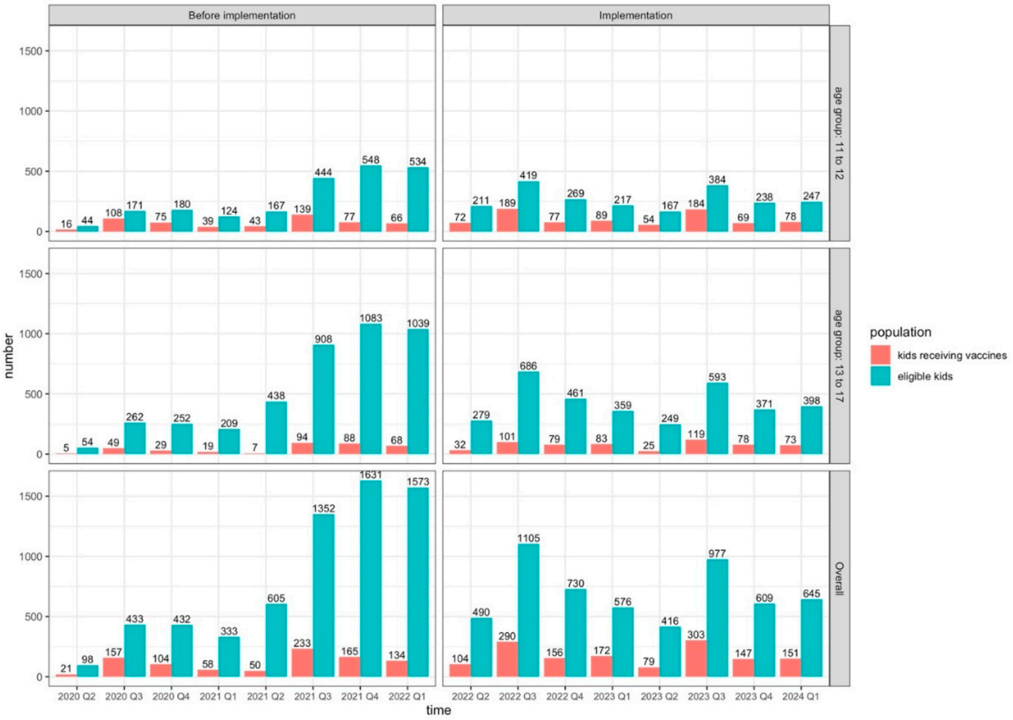

Supplementary Figure S4. Autocorrelation of vaccination volume.

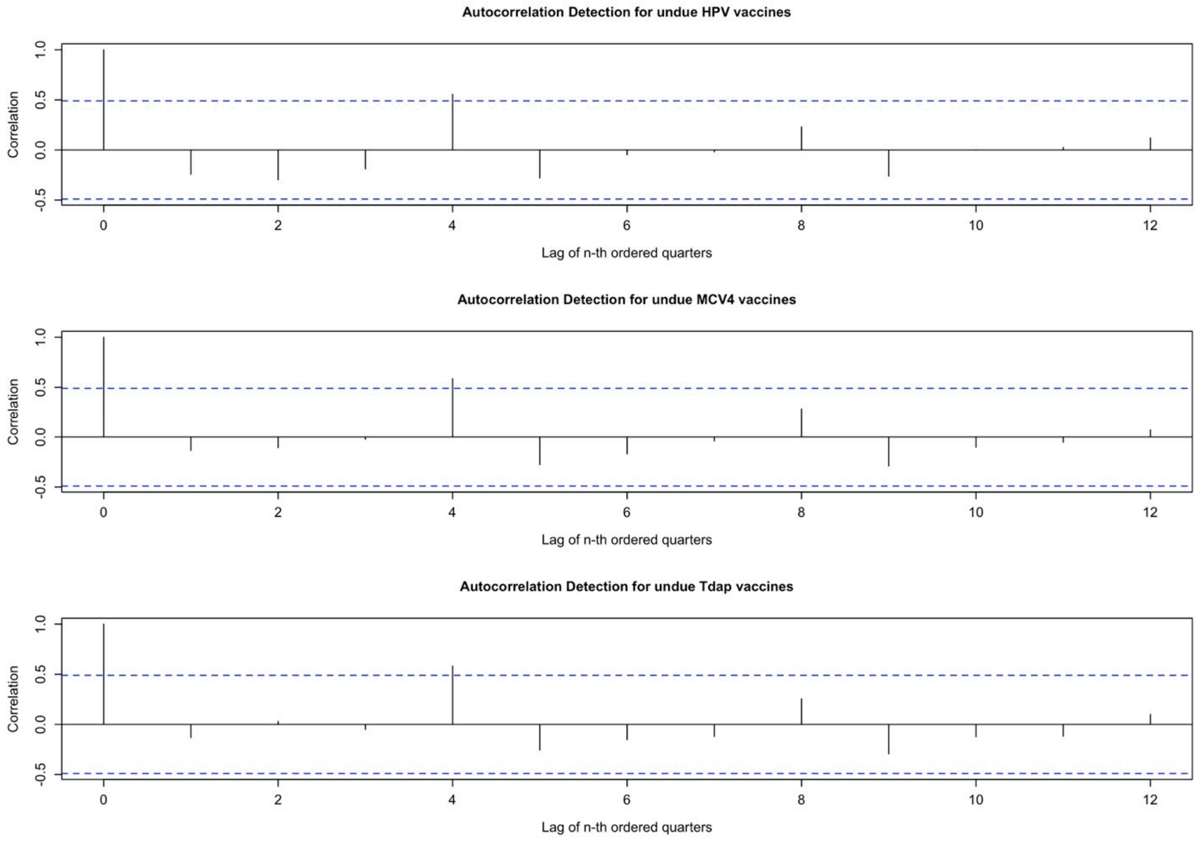

Autocorrelation of HPV, MCV4, Tdap quarterly vaccination volume was calculated by acf() in R stats package. Correlation greater than 0.5 indicates a potential autocorrelation at lag = n, except lag = 0 where correlation = 1 in default. All vaccinations demonstrate strong autocorrelation with lag = 4 quarter, indicating a strong annual pattern corresponding to the variations of initiation rates in Figure S3.

In Supplementary Figures S5–S7, we present model diagnostics by examining residual distributions overall and stratified by quarter (upper and lower panels, respectively). Residuals were approximately normally distributed across all models. Although minor quarter-specific dispersion was observed, no influential outliers were detected. It suggests the presence of some unmeasured variability; however, further model adjustment is constrained by the limited number of time points and available covariates. Overall, residual magnitudes were small. Therefore, the model fitting was still acceptable.

Supplementary Figure S5. Model diagnostic of HPV vaccination modelling.

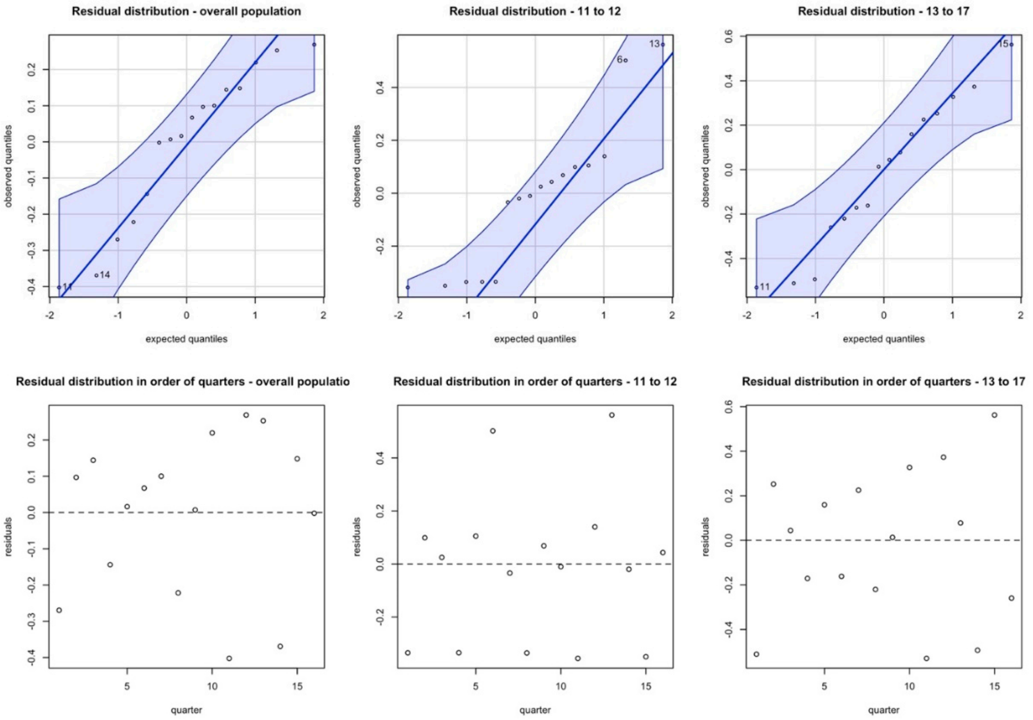

Supplementary Figure S6. Model diagnostic of MCV4 vaccination modelling.

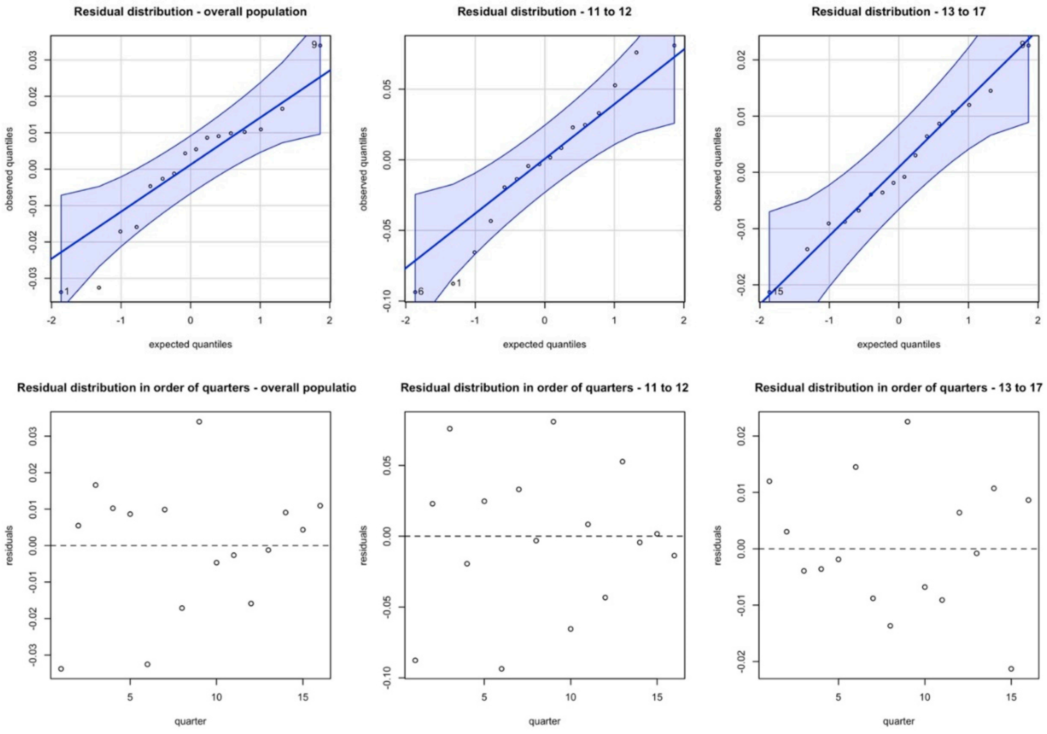

Supplementary Figure S7. Model diagnostic of Tdap vaccination modelling.

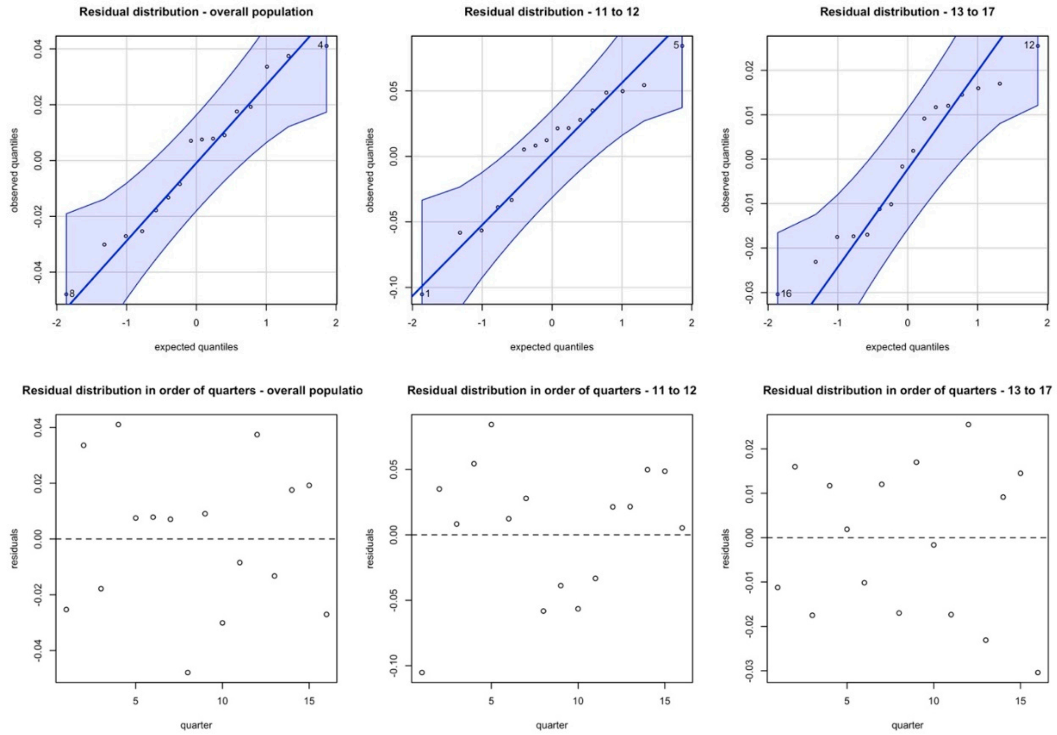

Supplement: Supplementary file 1 [file healthcare-14-00519-s001.zip › healthcare-4091660-supplementary.pdf]
